# Supplementary material for: Rapid and site-specific deep phosphoproteome profiling by data-independent acquisition without the need for spectral libraries
Source: Nat Commun. 2020 Feb 7;11:787. doi: 10.1038/s41467-020-14609-1 (PMC7005859; doi:10.1038/s41467-020-14609-1)
Supplement: Supplementary file 1 — Supplementary Information [file 41467_2020_14609_MOESM1_ESM.pdf]

Supplementary Information

Rapid and site-specific deep phosphoproteome profiling by data-independent acquisition without the need for spectral libraries

Bekker-Jensen et al.

Supplementary Figure 1

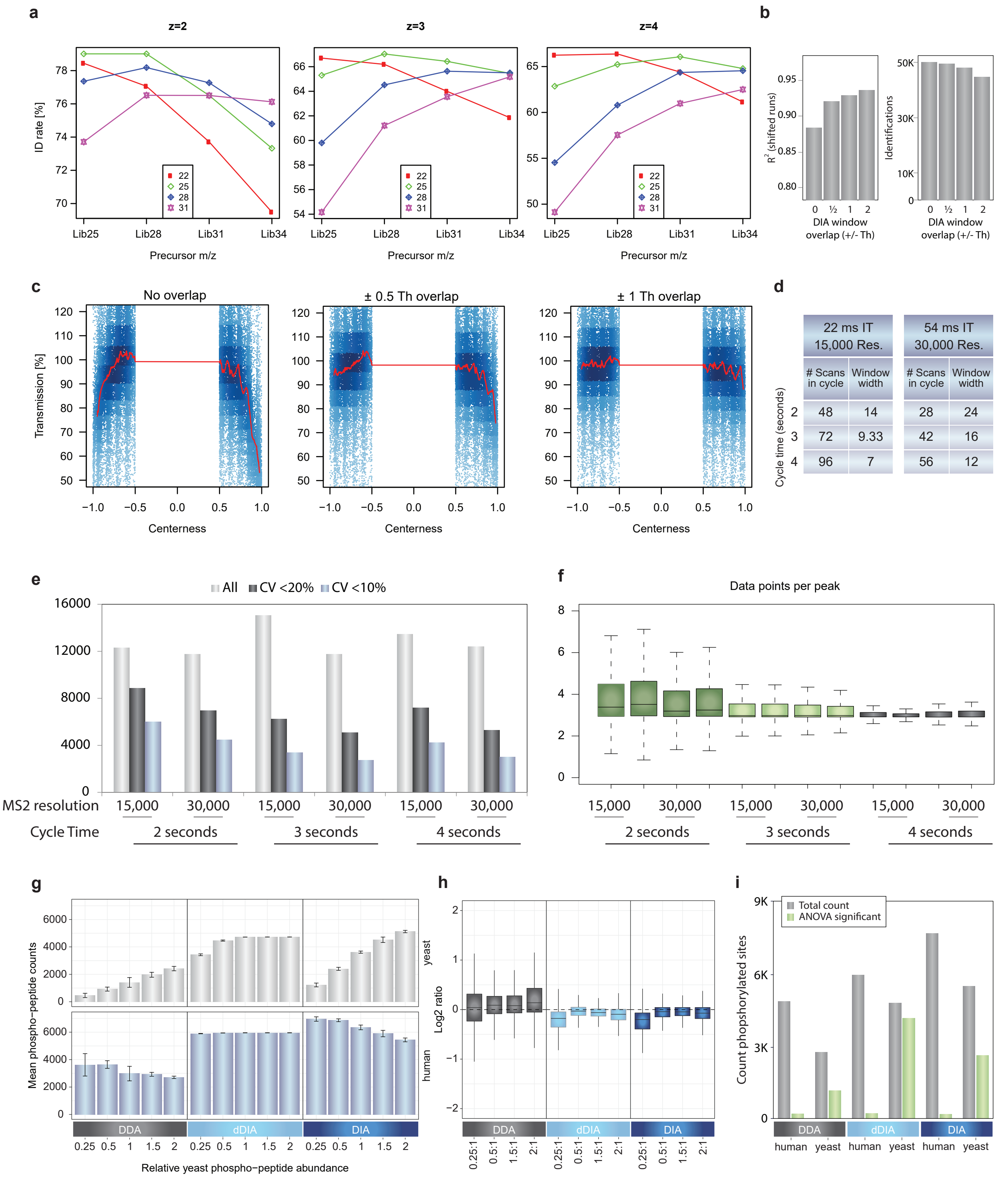

Supplementary Figure 1 High-throughput and sensitive phosphoproteomics for DDA & DIA - identification and quantification **(a)** Comparison of optimal NCE for spectral library generation and DIA runs **(b)** Identifications and correlation of IDs with different overlaps between windows **(c)** Comparison of overlap between mass windows **(d)** Schematic overview of methods used for cycle time optimization **(e)** Average identifications of phosphopeptides and the number of phosphopeptides with CVs below defined thresholds from duplicate measurements **(f)** Data points per peak for the different methods from duplicate measurements. Boxes mark the first and third quantile, with the median highlighted as dash, and whiskers marking the minimum/maximum value within 1.5 interquartile range. Outliers are not shown **(g)** Number of HeLa and yeast phosphopeptides measured with DDA, DIA and dDIA. Error bars represent mean ± SD of six independent measurements **(h)** Boxplot of measured and theoretical ratios for HeLa phosphopeptides with DDA & DIA from six independent measurements. Boxes mark the first and third quantile, with the median highlighted as dash, and whiskers marking the minimum/maximum value within 1.5 interquartile range. Outliers are not shown **(i)** Human and yeast significantly regulated phosphorylated sites by ANOVA from six indenpendent measurements. Source data for this figure are provided as a Source Data file.

Supplementary Figure 2

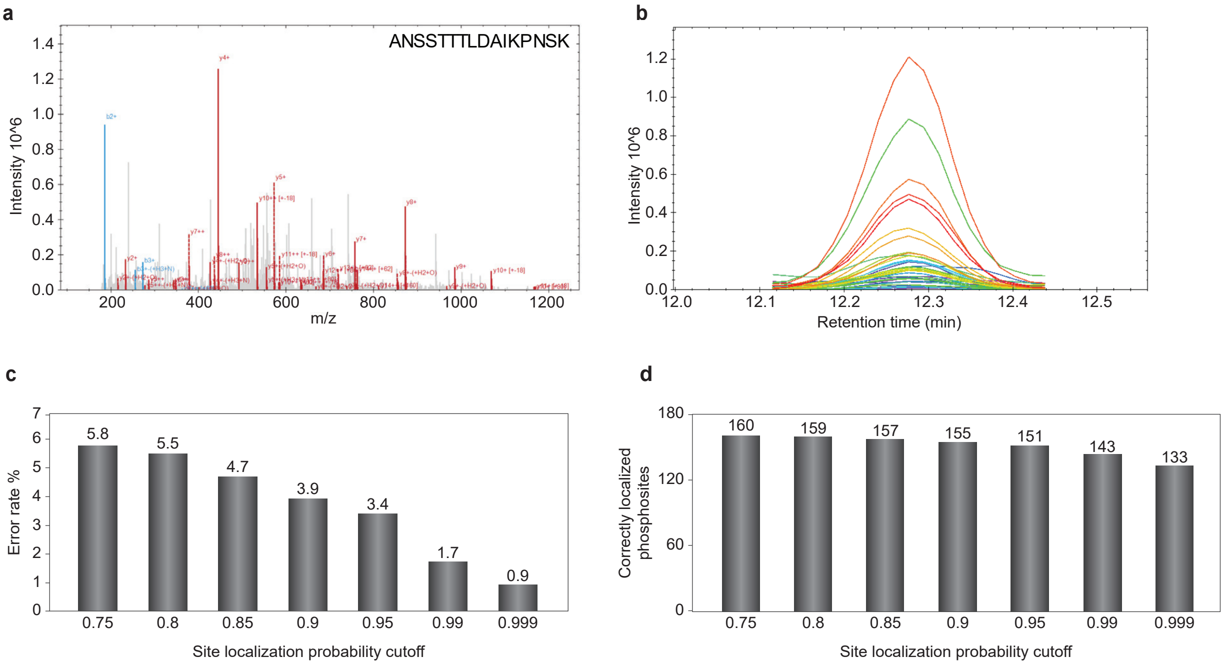

Supplementary Figure 2 PTM localization and error rate comparison for directDIA **(a)** Apex Fragment spectra and **(b)** extracted ion chromatogram for example peptide used in Supplementary table **(c)** Comparison of error rates at different probability cutoffs for dDIA from three independent measurement from three independent measurementss. **(d)** Comparison of correctly localized phosphosites at different probability cutoffs for dDIA from three independent measurements. Source data for this figure are provided as a Source Data file.

Supplementary figure 3

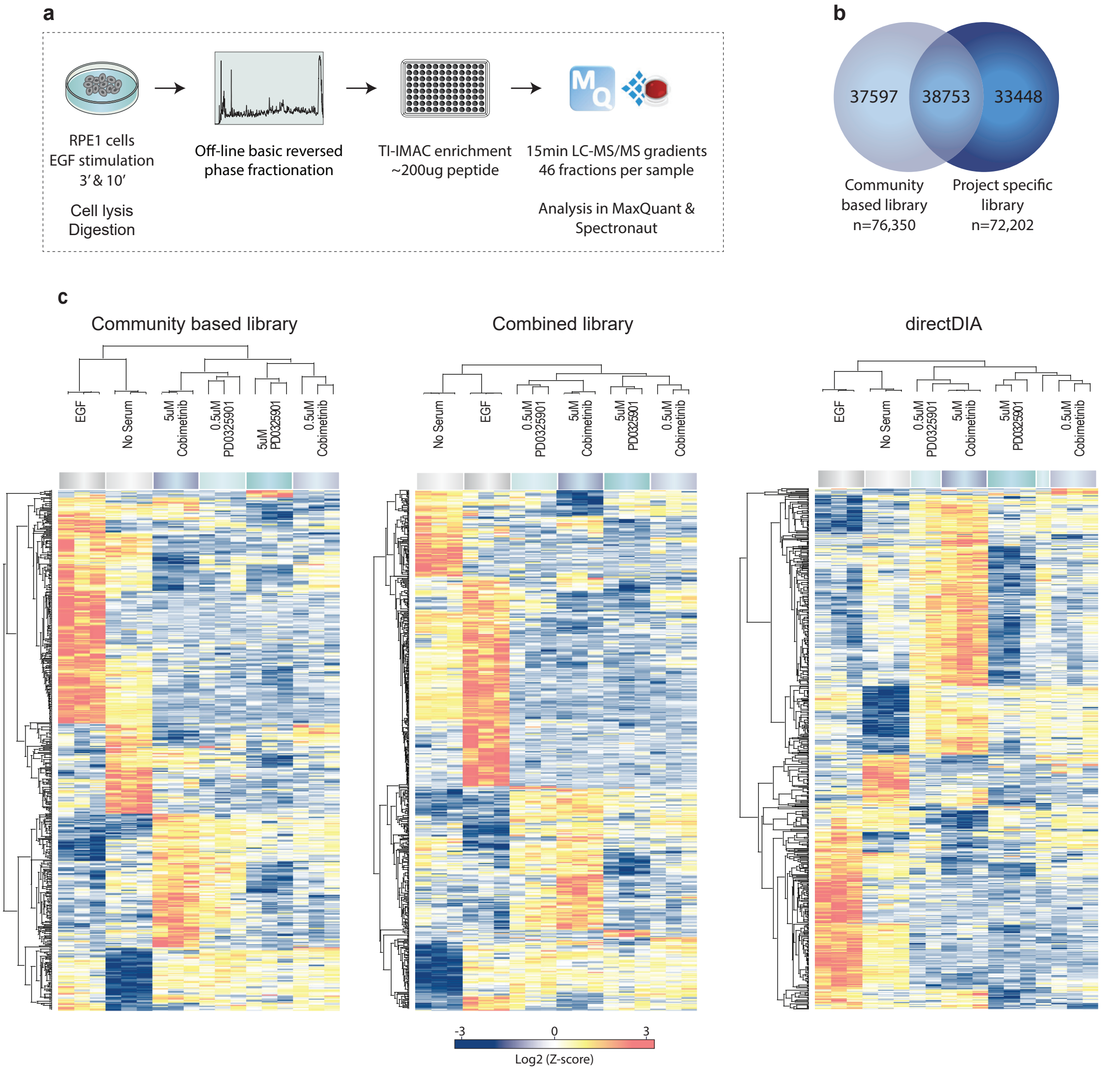

Supplementary figure 3 Technical comparison of DDA and different types of DIA in a biological setting **(a)** Experimental workflow for building project specific spectral library **(b)** Overlap of phosphopeptides between the project specific library and the community based library **(c)** Heatmap of unsupervised clustering analysis of ANOVA regulated phosphosites for DIA workflows with community based library, combined library and directDIA.

Supplementary figure 4

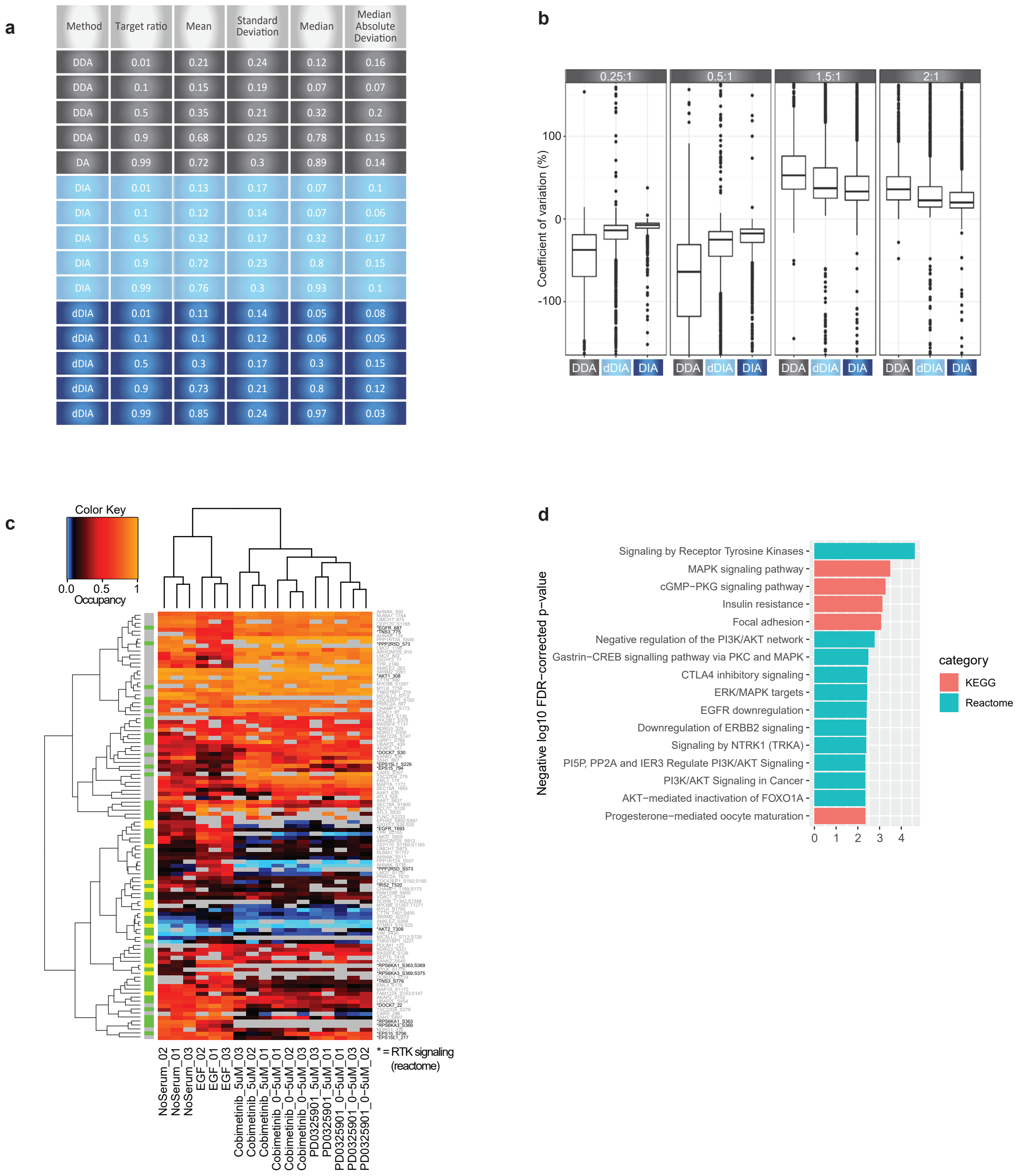

Method	Target ratio	Mean	Standard Deviation	Median	Median Absolute Deviation
DDA	0.01	0.21	0.24	0.12	0.16
DDA	0.1	0.15	0.19	0.07	0.07
DDA	0.5	0.35	0.21	0.32	0.2
DDA	0.9	0.68	0.25	0.78	0.15
DA	0.99	0.72	0.3	0.89	0.14
DIA	0.01	0.13	0.17	0.07	0.1
DIA	0.1	0.12	0.14	0.07	0.06
DIA	0.5	0.32	0.17	0.32	0.17
DIA	0.9	0.72	0.23	0.8	0.15
DIA	0.99	0.76	0.3	0.93	0.1
dDIA	0.01	0.11	0.14	0.05	0.08
dDIA	0.1	0.1	0.12	0.06	0.05
dDIA	0.5	0.3	0.17	0.3	0.15
dDIA	0.9	0.73	0.21	0.8	0.12
dDIA	0.99	0.85	0.24	0.97	0.03

Supplementary Figure 4 Stoichiometry benchmark **(a)** Overview of standard deviation and median absolute deviation for the different methods **(b)** Coefficent of variation for the different methods from six independent measurements. Boxes mark the first and third quantile, with the median highlighted as dash, and whiskers marking the minimum/maximum value within 1.5 interquartile range. Outliers are shown **(c)** Heatmap of ANOVA, FDR 0.20 regulated occupancies **(d)** Enrichment analysis of ANOVA significant occupancies. Source data for this figure are provided as a Source Data file.

Supplementary Note 1: Phospho-site algorithm

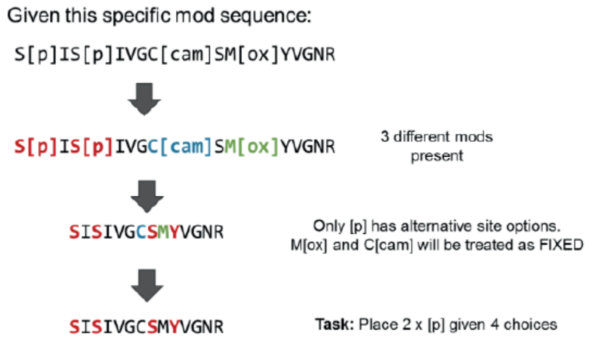

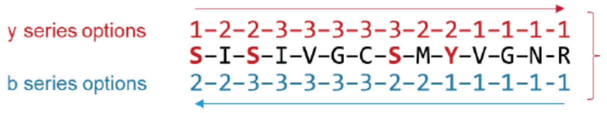

Only **56** DIFFERENT backbone ions across all 6 site candidates for “place 2 in 4”

SISIVGCSMYVGNR
SISIVGCSMYVGNR
SISIVGCSMYVGNR
SISIVGCSMYVGNR
SISIVGCSMYVGNR
SISIVGCSMYVGNR

Using the information we gathered from the previous processes, we can very efficiently generate now all possible site candidates for “place 2 in 4” using recursion.

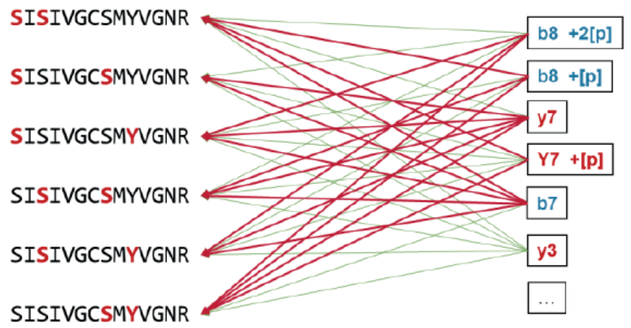

we not only assign the fragments that CONFIRM a specific site candidate, we also remember which fragments REFUTE those candidates

A feature is scored against the expected isotopic pattern (including penalty checks for mis-charged or mono-shifted features)

Feature score:

c = Correlation with expected pattern
I_{M0} = Intensity of the M0 peak
I_{M-1} = Intensity of the M-1 peak

$$S_{feature} = Max\left(0.0,\ c\ - Min\left(1.0, \frac{I_{m-1}}{I_{m0}}\right) - shiftScore\right)$$

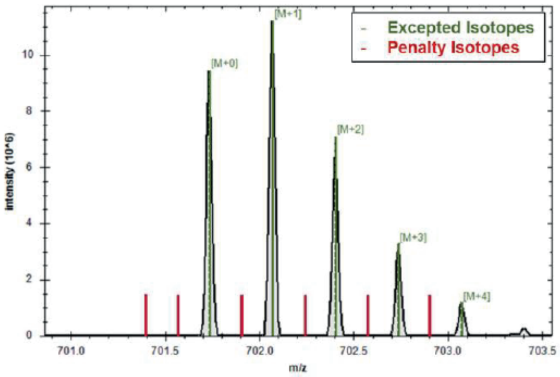

$$S_{feature} = Max\left(0.0,\ c - Min\left(1.0, \frac{I_{m-1}}{I_{mo}}\right) - shiftScore\right)$$

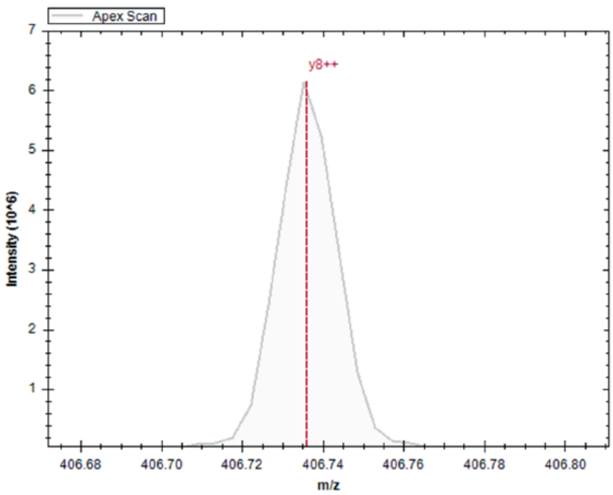

The shift score is calculated as the relative distance of the empirical m/z to the center of the tolerance window.

$$shiftScore = \frac{Abs(mz_{emp} - mz_{theo})}{tol}$$

Where mz_{emp} is the empirical m/z of this peak in the MS2 spectrum, mz_{theo} is the theoretical (calibrated) mz of the fragment and tol is the allowed mass tolerance (the maximum allowed shift).

A fragment perfectly in the center or the tolerance window would therefore have shift shiftScore of 0.0.

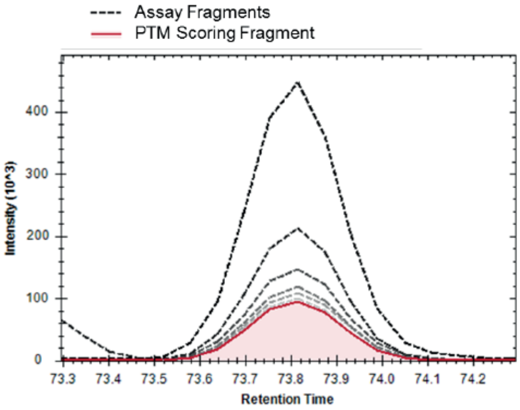

For all fragments, that achieved a feature score > 0.0 we now extract a short XIC corresponding to the peak start-end RT.

This short XIC is then correlated (Pearson correlation) against all fragment traces of the assay.

The median correlation against the assay is returned as the correlation score for this fragment.

$$ScoreFrg(frg) = Max\left(0.0, Log_{10}(frg_{Int}) * S_{feature}(frg) * S_{xic}(frg)\right)$$

A score for a single fragment MUST be positive and is calculated as a combination of the fragments apex intensity (frg_{Int}), the feature score and the XIC score.

We now only need to condense scores for all **CONFORMING** / **REFUTING** fragments of a given candidate into a final candidate score.

- $conf(cand) = \sum_j^{frgConf} ScoreFrg(j)$
- $refute(cand) = \sum_j^{frgRef} ScoreFrg(j)$
- $Score(cand) = e^{Max\left(1.0,\, conf(cand) * \left(\frac{cn}{cm}\right) - refute(cand) * \left(\frac{rn}{rm}\right)\right)}$

Where:

cn is the number of Fragments that where found and are confirming this site
cm is the total number of Fragments that would have confirmed this site
rn is the number of Fragments that where found and are refuting this site
rm is the total number of Fragments that would have refuted this site

Finally, we condense all the candidate scores into site probabilities by dividing the sum of all candidate scores that feature a specific site, by the sum of all candidate scores.

SISIVGCSMYVGNR $c_1 = Score(cand1)$

SISIVGCSMYVGNR $c_2 = Score(cand2)$

SISIVGCSMYVGNR $c_3 = Score(cand3)$

SISIVGCSMYVGNR $c_4 = Score(cand4)$

SISIVGCSMYVGNR $c_5 = Score(cand5)$

SISIVGCSMYVGNR $c_6 = Score(cand6)$

$$p(site) = \frac{(c_1 + c_2 + c_3)}{c_{all}}$$

$$C_{all} = \sum_{i=1}^{N(c)} c_i$$

$$\frac{(c_3 + c_5 + c_6)}{c_{all}}$$

Supplementary Note 2: Perseus plugin peptide collapse. This is a schematic of the data processing steps of the Perseus plugin "Peptide Collapse". The main purpose of the plugin is to combine Spectronaut (SN) precursor quantifications into consensus PTM sites or peptides. This should allow more robust statistical analysis, especially with missing values present in the data. Precursor collapse levels mimic the MaxQuant (MQ) site, evidence (= Modified sequence) and modification-specific-peptides output tables.

Localization probabilities:

R

Extract from…
- SN EG.PTMAssayProbability (= assay confidence)
- SN EG.PTMlocalizationProbabilities (= discovery confidence)
- MQ "… Probabilities" (from evidence.txt; e.g. "Phospho (STY) Probabilities")

- *or* - Ignore localization probabilities

↓

Precursor collapse:

PTM site-level

e.g. TP53_S15_M1

- Extract PTM positions from…
SN EG.PrecursorId
(= assay localization ; used for EG.PTMAssayProbability localization)
- or -
SN EG.PTMLocalizationProbabilities
(= discovery localization)

- PTM site-level expansion
(copy precursors per target PTM)

- Precursor grouping by conditions
(long-format to wide-format)

- Site-level precursor collapse
(Separated by multiplicities M1/2/>3)

PTM peptide-level

localized

e.g. S(ph)QETFS(ph)DLWK

- Extract PTM positions from…
SN EG.PrecursorId
(= assay localization ; used for EG.PTMAssayProbability localization)
- or -
SN EG.PTMLocalizationProbabilities
(= discovery localization)
- or -
MQ "Modified sequence"
(from evidence.txt)

- Precursor grouping by conditions
(long-format to wide-format)

- Localized peptide-level precursor collapse

non-localized

e.g. SQETFSDLWK_2(ph)

- Extract PTM positions from…
SN EG.PrecursorId
(= assay localization ; used for EG.PTMAssayProbability localization)
- or -
SN EG.PTMLocalizationProbabilities
(= discovery localization)

- Precursor grouping by conditions
(long-format to wide-format)

- Non-localized peptide-level precursor collapse

Summing

Intensities	**Condition 1**	**Condition 2**	**Condition 3**
Precursor 1	10,000	5000	-
Precursor 2	20,000	10,000	14,000
**Collapsed**	**30,000**	**15,000**	**14,000**

Summing: Precursor intensities are summed, disregarding missing values

PTM site-level:

- *Optional:*
Localization filtering (0 <= x <= 1)

- *Optional:*
Create PTM amino acid sequence window from FASTA file matching

PTM peptide-level, localized:

- *Optional:*
Localization filtering (0 <= x <= 1)

- *Optional:*
PTM stoichiometry calculation
(see *Hogrebe et al. Nat Commun 2018*)

PTM peptide-level, non-localized:

- *Optional:*
Localization filtering (0 <= x <= 1)
