# Supplementary material for: Rapid and site-specific deep phosphoproteome profiling by data-independent acquisition without the need for spectral libraries
Source: Nat Commun. 2020 Feb 7;11:787. doi: 10.1038/s41467-020-14609-1 (PMC7005859; doi:10.1038/s41467-020-14609-1)
Supplement: Supplementary file 3 — Description of Additional Supplementary Files [file 41467_2020_14609_MOESM3_ESM.docx]

**Description of Additional Supplementary Files**

**File Name: Supplementary Data 1**

**Description:** Benchmark of identification and quantification with Yeast/HeLa dilution

**File Name: Supplementary Data 2**

**Description:** Example of PTM localization

**File Name: Supplementary Data 3**

**Description:** Technical comparison of DDA and different types of DIA in a biological setting

**File Name: Supplementary Data 4**

**Description:** Comparison to Olsen et al., Cell 2006

**File Name: Supplementary Data 5**

**Description:** Stoichiometry benchmark

**File Name: Supplementary Data 6**

**Description:** Kinase inhibitor screen

**File Name: Supplementary Data 7**

**Description:** Known kinase substrates

**File Name: Supplementary Data 8**

**Description:** Roadmap to PRIDE archived files
