# Supplementary material for: Rapid and site-specific deep phosphoproteome profiling by data-independent acquisition without the need for spectral libraries
Source: Nat Commun. 2020 Feb 7;11:787. doi: 10.1038/s41467-020-14609-1 (PMC7005859; doi:10.1038/s41467-020-14609-1)
Supplement: Supplementary file 12 — Reporting Summary [file 41467_2020_14609_MOESM12_ESM.pdf]

nature research

Corresponding author(s): Jesper V. Olsen

Last updated by author(s): Jan 17, 2020

Reporting Summary

Nature Research wishes to improve the reproducibility of the work that we publish. This form provides structure for consistency and transparency in reporting. For further information on Nature Research policies, see Authors & Referees and the Editorial Policy Checklist .

Statistics

For all statistical analyses, confirm that the following items are present in the figure legend, table legend, main text, or Methods section.

n/a	Confirmed	
☐	☒	The exact sample size (*n*) for each experimental group/condition, given as a discrete number and unit of measurement
☐	☒	A statement on whether measurements were taken from distinct samples or whether the same sample was measured repeatedly
☐	☒	The statistical test(s) used AND whether they are one- or two-sided<br>*Only common tests should be described solely by name; describe more complex techniques in the Methods section.*
☐	☒	A description of all covariates tested
☐	☒	A description of any assumptions or corrections, such as tests of normality and adjustment for multiple comparisons
☐	☒	A full description of the statistical parameters including central tendency (e.g. means) or other basic estimates (e.g. regression coefficient) AND variation (e.g. standard deviation) or associated estimates of uncertainty (e.g. confidence intervals)
☐	☒	For null hypothesis testing, the test statistic (e.g. *F*, *t*, *r*) with confidence intervals, effect sizes, degrees of freedom and *P* value noted<br>*Give P values as exact values whenever suitable.*
☐	☒	For Bayesian analysis, information on the choice of priors and Markov chain Monte Carlo settings
☐	☒	For hierarchical and complex designs, identification of the appropriate level for tests and full reporting of outcomes
☐	☒	Estimates of effect sizes (e.g. Cohen's *d*, Pearson's *r*), indicating how they were calculated

Our web collection on statistics for biologists contains articles on many of the points above.

Software and code

Policy information about availability of computer code

Data collection	MaxQuant 1.6.5.0 and 1.6.0.17 and Spectronaut version 13.0.190309.20491
Data analysis	Transformation of the Spectronaut normal report and calculation of stoichiometry values was performed using a custom coded plugin “Peptide Collapse” in Perseus (1.6.5.0). The plugin was created using Microsoft Visual Studio 2017 (15.6.3) and requires Perseus and R (minimum version 3.6.0) to run. Detailed information on how to install and use the plugin and the calculations it performs are listed in Supplementary Note 1 and 2.

For manuscripts utilizing custom algorithms or software that are central to the research but not yet described in published literature, software must be made available to editors/reviewers. We strongly encourage code deposition in a community repository (e.g. GitHub). See the Nature Research guidelines for submitting code & software for further information.

Data

Policy information about availability of data

All manuscripts must include a data availability statement. This statement should provide the following information, where applicable:

- Accession codes, unique identifiers, or web links for publicly available datasets
- A list of figures that have associated raw data
- A description of any restrictions on data availability

Data are available via ProteomeXchange with identifier PXD014525 with the following Reviewer account details: Username: reviewer87979@ebi.ac.uk Password: ExL7jCEm. Password protection will be removed upon publication.

Field-specific reporting

Please select the one below that is the best fit for your research. If you are not sure, read the appropriate sections before making your selection.

- [x] Life sciences
- [ ] Behavioural & social sciences
- [ ] Ecological, evolutionary & environmental sciences

For a reference copy of the document with all sections, see nature.com/documents/nr-reporting-summary-flat.pdf

Life sciences study design

All studies must disclose on these points even when the disclosure is negative.

Sample size	3 technical or biological replicates were chosen.
Data exclusions	No data was excluded.
Replication	Biological replicates were carried out by harvesting cells on different passages and days. Downstream sample preparation were carreid out in parallel.
Randomization	Randomization was not carried out. All samples from one replicate were analyzed first and then the next replicate.
Blinding	Blinding was not carried out, since the technical strength of the method was the objective.

Reporting for specific materials, systems and methods

We require information from authors about some types of materials, experimental systems and methods used in many studies. Here, indicate whether each material, system or method listed is relevant to your study. If you are not sure if a list item applies to your research, read the appropriate section before selecting a response.

Materials & experimental systems

n/a	Involved in the study	
x		Antibodies
	x	Eukaryotic cell lines
x		Palaeontology
x		Animals and other organisms
x		Human research participants
x		Clinical data

Methods

n/a	Involved in the study	
x		ChIP-seq
x		Flow cytometry
x		MRI-based neuroimaging

Eukaryotic cell lines

Policy information about cell lines

Cell line source(s)	RPE1-hTert, HeLa
Authentication	Cell lines were authenticated by STR profiling using the ATCC authentication service according to manufacturers' instructions.
Mycoplasma contamination	Cell lines have been tested negative for mycoplasma.
Commonly misidentified lines (See ICLAC register)	N/A
